# A Feasibility Study with Image-Based Rendered Virtual Reality in Patients with Mild Cognitive Impairment and Dementia

**DOI:** 10.1371/journal.pone.0151487

**Published:** 2016-03-18

**Authors:** Valeria Manera, Emmanuelle Chapoulie, Jérémy Bourgeois, Rachid Guerchouche, Renaud David, Jan Ondrej, George Drettakis, Philippe Robert

**Affiliations:** 1 EA CoBTeK / IA, University of Nice Sophia Antipolis, Nice, France; 2 Institut National de Recherche en Informatique et en Automatique, Sophia Antipolis, France; 3 Centre Mémoire de Ressources et de Recherche, CHU de Nice, Nice, France; 4 Disney Research Los Angeles, Los Angeles, California, United States of America; 5 Trinity College Dublin, Dublin, Ireland; University of California, San Francisco, UNITED STATES

## Abstract

Virtual Reality (VR) has emerged as a promising tool in many domains of therapy and rehabilitation, and has recently attracted the attention of researchers and clinicians working with elderly people with MCI, Alzheimer’s disease and related disorders. Here we present a study testing the feasibility of using highly realistic image-based rendered VR with patients with MCI and dementia. We designed an attentional task to train selective and sustained attention, and we tested a VR and a paper version of this task in a single-session within-subjects design. Results showed that participants with MCI and dementia reported to be highly satisfied and interested in the task, and they reported high feelings of security, low discomfort, anxiety and fatigue. In addition, participants reported a preference for the VR condition compared to the paper condition, even if the task was more difficult. Interestingly, apathetic participants showed a preference for the VR condition stronger than that of non-apathetic participants. These findings suggest that VR-based training can be considered as an interesting tool to improve adherence to cognitive training in elderly people with cognitive impairment.

## Introduction

Due to the risen average life-span, we are witnessing a dramatic increase of the incidence of age-related disorders such as dementia, a decline in mental ability severe enough to interfere with activities of daily living [1]. Dementia can result from different causes, the most common being Alzheimer's disease (AD), and it is often preceded by a pre-dementia stage, known as Mild Cognitive Impairment (MCI), characterized by a cognitive decline greater than expected for an individual’s age, which however does not interfere notably with activities of daily living [2,3]. Dementia and MCI are characterized by the presence of cognitive symptoms, such as impaired memory, attention, orientation and executive functions, which are often associated with behavioral and psychological symptoms, one of the most common being apathy, a disorder of motivation [4–6]. Attentional problems are very common in patients with MCI and dementia (e.g., [7–9]). Current evidence suggests that after an initial amnesic stage in Alzheimer's disease, attention is the first non-memory domain to be affected, before deficits in language and visuospatial abilities [10]. In addition, attention and motivation share a common neural network, involving the cingulate, dorsolateral prefrontal and inferior parietal cortices [11]. For these reasons, attentional deficits are considered of particular interest in the assessment and rehabilitation of these patients.

In the last decades, many promising disease-modifying treatments for AD and related disorders have been proposed. However, clinical trials conducted on the treatments’ efficacy did not lead to important breakthroughs, thus resulting in a growing interest in the domain of non-pharmacological treatments [12]. Participation in stimulating mental activities at older age may represent a protective factor against cognitive decline, possibly reducing the risk of developing dementia [13]. Cognitive training based on computerized tasks and exercises represent a promising solution to engage participants in structured mental activities and enhance their cognitive functions, especially if they incorporate a motivational, playful aspect [14]. This is the case of Serious Games, which are digital applications specialized for purposes other than entertainment, such as educating, informing, or enhancing cognitive and/or physical functions. Recent meta-analyses demonstrated the efficacy of cognitive training on primary cognitive outcomes in cognitively healthy adults [15] and participants with MCI [13]. In addition, cognitive games (i.e., games which target cognitive improvement) are considered interesting and motivating by patients with MCI and AD (e.g., [16]), and have been shown to improve a number of cognitive functions in those patients, such as attention and memory [17–19] and visuo-spatial abilities [20] (see [21] for a review). Although preliminary, these results suggest that ICT-based cognitive training (that is cognitive training based on Information and Communication Technologies) is useful both in primary prevention (i.e., to reduce disease incidence in cognitively normal individuals) and secondary prevention (i.e., to slow the progression of pre-clinical disease to clinical disease). Evidence of the effectiveness of cognitive exercise as a tertiary prevention tool (i.e., to reduce the disability due to cognitive symptoms in diagnosed patients) is still sparse, but promising (e.g., [22]).

Recently, cognitive training based on immersive Virtual Reality (VR) has attracted the attention of clinicians and researchers in the field of MCI and dementia, and has emerged as a promising tool in many domains of therapy and rehabilitation [23]. Successful applications have been developed for the treatment of phobias, stress, anxiety, as well as for post-stroke rehabilitation and pain mitigation [23–24]. VR has several advantages compared to classical paper-pencil tasks in designing effective cognitive trainings. Just to mention some, VR has an enhanced ecological validity, that is, a higher degree of similarity between the training environment and the real world; this is supposed to represent an added value for predicting an improvement in everyday functioning. Second, a VR setting–as any computerized test—offers the possibility to provide immediate performance feedback, which is generally accepted to be necessary for most forms of learning and for successful rehabilitation. Finally, VR offers the possibility to personalize the environment and the activities, to make them more engaging. Persons with dementia are more engaged by stimuli and activities that match their interests and personal history [25–27]; as engagement (the act of being occupied or involved with an external stimulus) has been shown to have positive effects on quality of life and functional abilities [28–29], VR has good potentials for designing successful trainings in these populations. Fully immersive VR systems consist of 3D displays that virtually place the patient inside the virtual environment for the highest level of immersion. Only a few studies so far employed immersive VR in patients with MCI and AD. Most of these VR solutions are designed for assessment purposes (e.g., [30–31]), but a few applications have been developed for training purposes (see [24] for a review). For instance, Optale and colleagues [32] designed a VR memory stimulation training based on the delivery of auditory stimuli and musico-therapy. The results of a Randomized Control Trials conducted on healthy elderly participants showed a significant improvement in memory tests and in several other aspects of cognition in participants who received the intervention compared to those who received a classical musico-therapy intervention. In the context of the European FP7 project VERVE (Vanquishing Fear through e-inclusion, http://verveconsortium.eu/), we recently developed a fully immersive VR application based on image-based rendering [33] allowing participants to navigate in well-known environments of their city, which was employed in reminiscence therapy. Virtual animated humans were present in the Virtual Environments [34–35]. A feasibility study conducted on healthy elderly participants showed that the task and environment were rated as motivating, and that they could represent a useful tool to improve reminiscence in a laboratory setting [36–37].

The purpose of the present work was to extend the experience gained in that project to clinical settings involving patients with MCI and dementia. Specifically, following similar technical procedures, we developed an image-based rendered environment to train selective and sustained attention. Our task is based on the principles of the classical cancellation task, employed for instance in the Attention Process Training—an intervention designed to rehabilitate attentional problems in people with brain injuries [38]. In the cancellation tasks [39–41], participants have to select targets among similar distractors. Here, participants had to find target virtual characters wearing special T-shirts embedded in a crowd of similar characters, placed in a well-known location of Nice (France), the city where the study took place.

In the present article, we describe a feasibility study conducted on patients with MCI and dementia, with or without apathy with this VR task, in order to evaluate the acceptability, interest and eventual usability problems in this population. Based on the results of previous studies performed with healthy elderly participants [36], we hypothesized that the VR setting would be acceptable and interesting for persons with MCI and dementia, and that it would be preferred over a classical paper-pencil task assessing the same abilities.

## Methods

### Participants

Patients were recruited at the Nice Research Memory Center & CoBTeK research unit (CMRR), located at the Institut Claude Pompidou. Thirty participants with MCI and 30 participants with dementia volunteered to take part in the study. Three participants (two participants with MCI and one with dementia) were excluded from the data analysis because they did not meet all the inclusion criteria (two participants because the MMSE score was not in the correct range, one because she presented major perceptual impairments; see below), leaving the final sample to 28 participants with MCI (13 female and 15 male; mean age = 75.0 years; SD = 6.8; age range = 62–89) and 29 patients with dementia (12 female, 17 male; mean age = 76.3 years; SD = 7.2; age range = 65–90). Patients with dementia included 15 patients with AD, 10 patients with mixed dementia, one patient with vascular dementia, two patients with primary progressive aphasia (PPA) and one patient with organic brain syndrome (OBS) according to the International Classification of Diseases 10^th^ revision (ICD 10). MCI diagnosis was conducted according to the National Institute on Ageing and Alzheimer Association group clinical criteria [42]. The Mini Mental State Exam (MMSE) was used to evaluate the level of cognitive impairment for each group [43]. Participants were included in the study if they had a MMSE score between 16 and 28. Participants were not included if they were younger than 60 years, if they had psychiatric disorders, major perceptual (visual or auditory) impairments, suffered from migraine or epilepsy, were motion-sickness sensitive, or were unable to provide informed written consent due to severe cognitive impairment.

Characteristics of MCI and dementia subjects are presented in Table 1. The age, level of education and gender distribution were not significantly different between the two groups. All participants provided their informed written consent before beginning the study. The study was performed in compliance with the Declaration of Helsinki, and was approved by the following ethics committees: Comité de Protection des Personnes (CPP)—Sud Méditerranée V, and Agence Nationale de Sécurité du Médicament et des Produits de Santé (ANSM).

**Table 1:** Characteristics and group comparisons for participants with MCI and dementia.

	MCI (N = 28)	Dementia (N = 29)	P
Female, n (%)	13 (46.4%)	12 (41.4%)	.453
Age (years), mean ± SD	75.0 ± 6.7	76.3 ± 7.2	.473
Level of education, n (%)			.778
Unknown	1 (3.6%)	0 (0%)	
No formal education	1 (3.6%)	0 (0%)	
Primary education	10 (35.7%)	9 (31.0%)	
Secondary education (first cycle)	5 (17.9%)	6 (20.7%)	
Secondary education (second cycle)	3 (10.7%)	4 (13.8%)	
Higher education	8 (28.6%)	10 (34.5%)	
MMSE, mean ± SD	25.4 ± 2.6	20.2 ± 3.1	.000*
CDR (sum of boxes)	1.4 ± 1.5	4.8 ± 3.1	.000*
Presence of Diagnostic Criteria for Apathy, n (%)	3 (10.7%)	20 (69.0%)	.000*
Apathy Inventory, mean ± SD	0.9 ± 1.8	4.0 ± 2.2	.000*

Legend. Group comparisons were made using ANOVAs, and chi-square for categorical testing.

* p < .001

### Materials and Procedure

**Fig 1:**
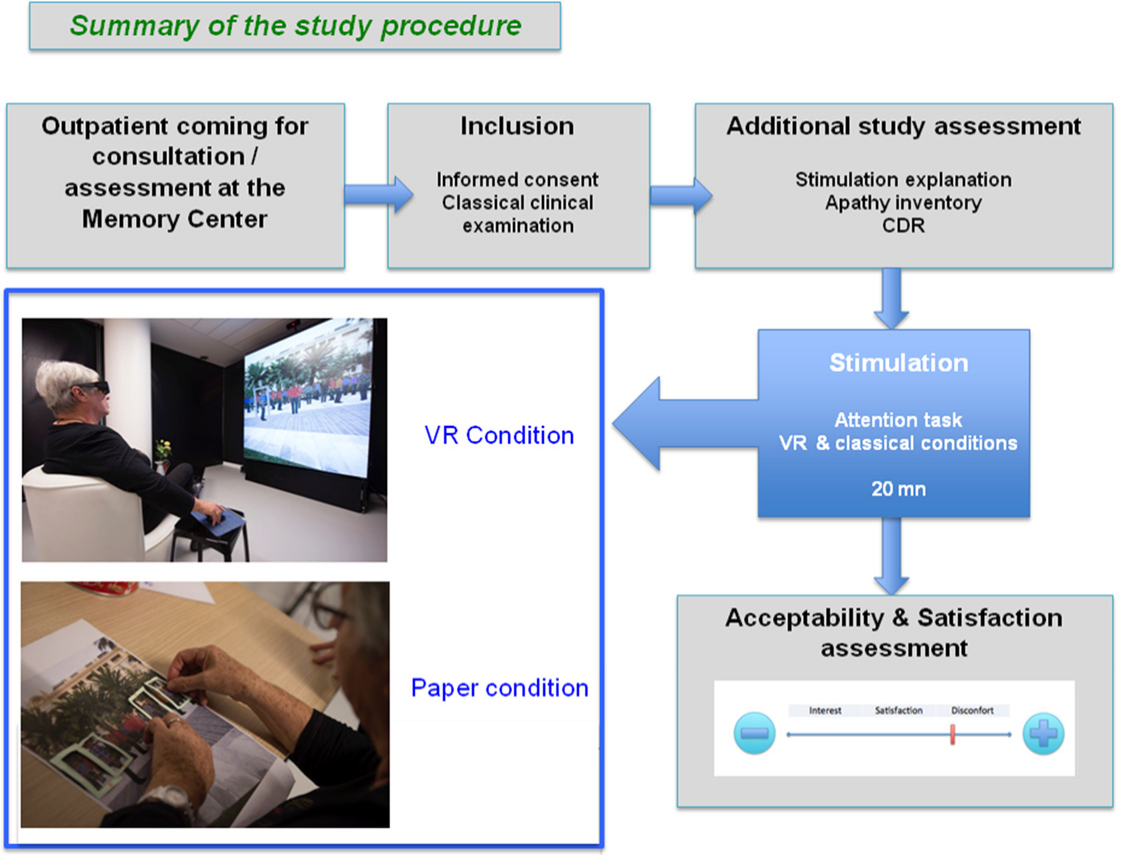
Summary of the study procedure.

Patients coming to the Nice Research Memory Center for a regular medical consultation or a classical neuropsychological assessment, if eligible, were invited to take part in the study. The inclusion period lasted three and a half months. If interested, they were asked to read and sign the informed written consent and follow the study procedure. They performed the study attentional task in two conditions: a paper condition, and a VR condition, presented in a randomized order. After each condition, participants were asked to fill in self-report questionnaires concerning their game experience. After the end of the second condition, they were asked which condition they preferred, and were told that they could continue playing for a few minutes, if they wished and if they had time. A summary of the study procedure can be found in Fig 1.

#### Cognitive and Behavioral assessment

Global cognitive and behavioral functioning was performed following the standard assessment used in the center, including the MMSE and the CDR-SOB, Sum Of Boxes scores [44]. The presence of apathy was evaluated by means of the diagnostic criteria for apathy [45], and the criteria have been used to divide the population in apathetic versus non-apathetic subjects. In addition, the severity of apathy was assessed using the Apathy Inventory–clinician version [6], a 12-point scale evaluating the presence of reduced initiation, interest and emotional blunting.

#### Attention task

Similarly to the classical cancellation tests employed to assess selective and sustained attention [39–41], participants were asked to find and select targets surrounded by distracters. Targets consisted of human female characters wearing T-shirts responding to specific criteria (N = 5), while distractors consisted of the same female characters wearing different T-shirts (N = 20). We defined three search criteria, corresponding to three levels of task difficulty:

*Color criterion*. Each character wore a T-shirt in one of the following 7 solid colors: red, orange, yellow, green, blue, purple, or grey. For each scene, five characters wore T-shirts in the colors defined as targets, while the remaining characters wore T-shirts in non-target colors.

*Pattern criterion*. Each character wore a T-shirt with a grey background and one of the following 7 white patterns: triangles, circles, squares, diamonds, horizontal stripes, horizontal grid, and oblique grid. For each scene, five characters wore T-shirts with the patterns defined as targets, while the remaining characters wore T-shirts with non-target patterns.

**Fig 2:**
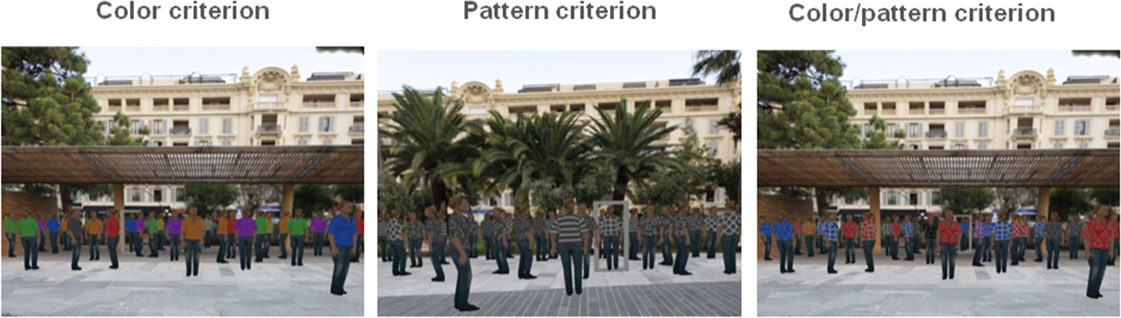
Examples of stimuli employed in the attentional task. Participants were asked to find five characters corresponding to specific criteria among a crowd of distracters.

*Color/Pattern criterion*. Each character wore a colored T-shirt (in one of the 7 colors employed in the color criterion scenes) with a white pattern (one of the 7 patterns employed in the pattern criterion scenes). For each scene, five characters wore T-shirts with the colors and patterns defined as targets, while the remaining characters wore T-shirts with different combinations of colors and patterns (Fig 2).

The scene where the characters were displayed was a well-known square in the city of Nice (France), and was rendered using image-based rendering, a computer graphics technique consisting in reconstructing a 3D environment from a set of photographs [33]. Synthetic 3D characters were displayed on top of the environment layer and were animated, slowly moving their arms [34–35].

*Procedure*. Participants were asked to play for five minutes in both the VR and the paper condition, presented in a randomized order. For each scene, participants were asked to select the five target characters. Scenes were presented in the following order: two color criterion scenes (total N of targets = 10), three pattern criterion scenes (total N of targets = 15), and three color/pattern criterion scenes (total N of targets = 15). If participants completed all these levels in five minutes, they were presented with additional color/pattern criterion scenes. Participants were allowed to progress to the next scene only after all the five targets had been found. The experimenter took note of the number of errors (incorrect selection of non-target characters) and number of targets found.

*Virtual reality condition (VR)*. Stimuli were displayed on a Barco OverView OLSF-721 full HD 3D stereoscopic LED video wall with a resolution of 1920 x 2160 pixels, and dimensions of 1.55 x 1.74 meters. Participants sat on a comfortable chair, at a distance of approximately 1.90 meters from the screen, and wore Volfoni Edge 1.2 active 3D LCD shutter glasses, synchronized with a Volfoni ActivHub IR100 infrared emitter. The computer running the program was equipped with an NVIDIA Quadro 6000 graphics card. Participants interacted with the VR application using a wireless mouse which was placed on a small stool next to the chair, so that the users’ arms were in a resting pose. Participants were asked to use the mouse to move a grey rectangle shape over the target character, and to click a mouse button to select the character. When a character was correctly selected, a green rectangle shape appeared over the character, while if the selection was incorrect, a red rectangle shape appeared over the character. The green (or red) shape remained on a character once she had been selected, in order to remind participants which characters had been found. Before starting the task, participants were presented with one practice color scene, in order to familiarize them with the task and materials (e.g., the mouse).

*Paper condition*. Screenshots of the 2D version of the VR scenes were printed on A3 paper sheets. Participants sat on a comfortable chair, in front of a desk with the paper sheets. They were asked to select the characters by placing green rectangle shapes over them. The shape remained on the character once she had been selected, in order to remind participants which characters had been found. Before starting the task, participants were presented with one practice color scene, in order to familiarize them with the task.

#### Self-report questionnaires

At the end of each experimental condition, participants were administered self-report questionnaires concerning the evaluation of their experience. Specifically, participants were presented with 10 cm analog scales adapted from Manera and colleagues [16] and Benoit and colleagues [36], and asked to report their level of satisfaction, interest, discomfort, anxiety, feeling of security and fatigue by bisecting a line, ranging from ‘not at all’ to ‘extremely’.

### Statistical analysis

Statistical analysis was computed using SPSS 20.0. In order to verify the acceptability of the intervention, we computed: a) the mean scores for the self-report questionnaires, separately for the VR and the paper condition; b) the number and percentage of participants who reported to have preferred each of the two conditions, and, at the exploratory level, c) the number of participants who continued playing after the experiment, along with the additional time played for each condition. Task performance in each condition was assessed by means of the number of targets correctly found in five minutes, and by the number of errors (non-target stimuli incorrectly selected). Group comparisons were performed with repeated-measures ANOVAs with Condition (VR vs. paper) as within-subject factor and Diagnosis (MCI vs. dementia) as between-subject factor. When comparing small groups (e.g., participants who continued playing after the experiment), between-subject ANOVAs were replaced by non-parametric Mann-Whitney U tests, and within-subject comparisons were performed by means of Wilcoxon signed-rank tests.

In order to explore the effects of the presence of diagnostic criteria for apathy, as participants were not balanced across groups, we performed Mann-Whitney U tests with Presence of diagnostic criteria for apathy (yes vs. no) as between-subject factor. Due to the exploratory nature of the analyses, we adopted a liberal criterion and did not apply any correction for multiple comparisons.

## Results

### Cognitive and behavioral assessment

Demographic, cognitive and behavioral characteristics of the patients are presented in Table 1 (see also S1 Dataset). Compared to MCI participants, participants with dementia had significantly lower MMSE scores (F_(1,55)_ = 47.51, p < .001, partial η^2^ = .46) and significantly higher CDR-SOB scores (t_(1,53)_ = 24.60, p < .001, partial η^2^ = .32), confirming the presence of a significant level of cognitive impairment, and a significant impairment in the activities of daily living. Also, participants with dementia had a higher Apathy Inventory compared to MCI participants (t_(1,55)_ = 34.64, p < .001, partial η^2^ = .39), and, compared to MCI participants, a higher proportion of participants met the diagnostic criteria for the presence of apathy (χ^2^ = 20.08, p < .001). The non-parametric Mann-Whitney U tests comparing apathetic and non-apathetic patients revealed that, for the MCI group, there was no difference between apathetic participants (N = 3) and non-apathetic participants (N = 25) in the CDR-SOB score (apathetic participants, M = 3.5, SD = 3.5, non-apathetic participants, M = 1.3, SD = 1.2; p = 222), MMSE score (apathetic participants, M = 24.0, SD = 2.0, non-apathetic participants, M = 25.6, SD = 2.6; p = .192) and age (apathetic participants, M = 71.7, SD = 10.7, non-apathetic participants, M = 75.4, SD = 6.4; p = .477). For the dementia group, no difference was found between apathetic (N = 20) and non-apathetic patients (N = 9) in the CDR-SOB (apathetic participants, M = 5.4, SD = 3.3, non-apathetic participants, M = 3.4, SD = 2.3; p = .116), MMSE score (apathetic participants, M = 20.7, SD = 3.2, non-apathetic participants, M = 19.2, SD = 2.8; p = .295) and age (apathetic participants, M = 74.7, SD = 6.9, non-apathetic participants, M = 80.1, SD = 6.8, p = .085). A significant inverse correlation between age and MMSE was found (r_(27)_ = -.44, p = .017).

### Intervention acceptability

#### Self-report questionnaire

**Fig 3:**
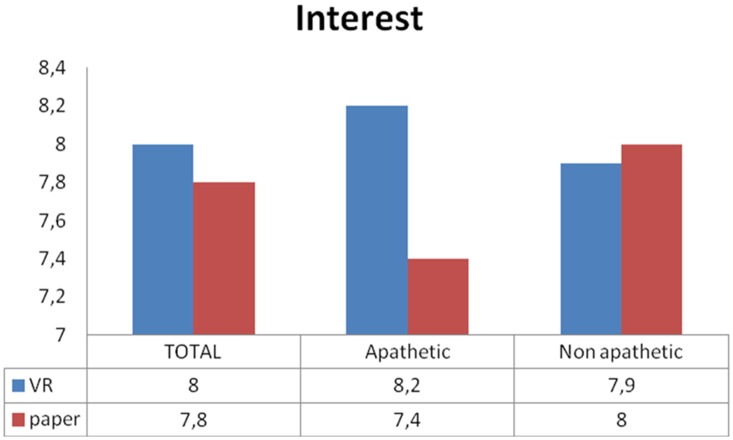
Self-report questionnaires. Mean scores for the self-report questionnaires in the Virtual Reality (VR, blue) and the classical paper version (red) conditions. Rating scale: 0 to 10. *p < .05 in the ANOVA.

Results of the self-report questionnaires for the VR and the paper conditions are presented in Fig 3 (see also S1 Dataset).

**Fig 4:**
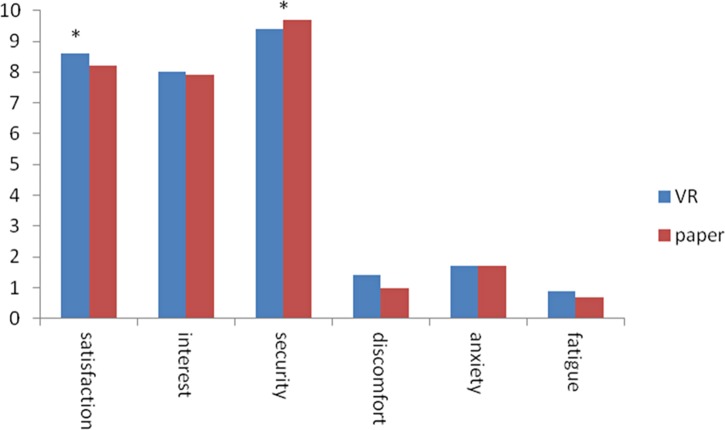
Results of the self-reported interest for apathetic and non-apathetic participants. Results of the self-reported interest for the Virtual Reality condition (VR, blue) and the classical paper condition (red) for the total sample, apathetic participants and non-apathetic participants.

For both the VR and the paper conditions, participants reported to be highly satisfied concerning the experience (VR, M = 8.6/10, SD = 1.7; paper: M = 8.2/10, SD = 2.3) and highly interested (VR, M = 8.0/10, SD = 2.3; paper: M = 7.9/10, SD = 2.2). They reported high feelings of security in both conditions (VR, M = 9.4/10, SD = 1.3; paper: M = 9.7/10, SD = 1.1). Furthermore, participants reported low levels of discomfort (VR, M = 1.4/10, SD = 2.5; paper: M = 1.0/10, SD = 1.9), anxiety (VR, M = 1.7/10, SD = 2.9; paper: M = 1.7/10, SD = 3.2), and fatigue (VR, M = .9/10, SD = 2.0; paper: M = .7/10, SD = 1.8) in both conditions. Results were computed taking into account the diagnosis and presence of diagnostic criteria for apathy. A repeated-measures ANOVA with Condition (VR vs. paper) as within-subject factor and Diagnosis (dementia vs. MCI) as between subject factor revealed the presence of a main effect of Condition only for the satisfaction (F_(1,55)_ = 4.42, *p* = .040, partial η^2^ = .07) and the security (F_(1,55)_ = 7.85, *p* = .007, partial η^2^ = .13) rating questions. Specifically, participants were significantly more satisfied in the VR condition compared to the paper condition, and that they felt less secure in the VR condition compared to the paper condition. No other comparison reached statistical significance (all ps > .234). No main effect of Diagnosis or interaction between Condition and Diagnosis was found in any of the rating scales (all ps > .254). Concerning the comparison between apathetic and non-apathetic participants, the Mann-Whitney U test revealed no effect of Presence of diagnostic criteria for apathy in any of the rating scales (all ps > .188). Interestingly, Mann-Whitney U tests on the difference between VR condition and paper condition (run to test for interactions between Condition and Presence of diagnostic criteria for apathy) revealed a significant effect of the Presence of diagnostic criteria for apathy for the interest question (p = .002), with apathetic participants significantly more interested in the VR condition compared to the paper condition than non-apathetic participants (see Fig 4). No other comparison reached statistical significance (all ps > .166).

#### Preference and additional time played

At the end of the experiment, participants were asked to decide which condition they had preferred, and they were free to continue playing, if they wished and had time. 39 participants (68.4%) reported that they had preferred the VR condition, 15 participants (26.3%) reported to have preferred the paper condition, and 3 participants (5.3%) expressed no preference. Reasons reported for the preference of the VR condition included: more engaging experience, more motivating setup, more immersive experience. Reasons reported for the preference of the paper condition included: easier setup (no mouse), more familiar experience, and less tiring for the eyes.

Out of the 57 participants, 12 (21.1%) continued playing after the end of the study, 9 in the VR condition (five participants with MCI and four with dementia; 7 non-apathetic and two apathetic participants), and three (three participants with dementia, all apathetic) in the paper condition (see Table 2). Wilcoxon signed-rank test revealed no main effect of Condition (*p* = .158). Similarly, Mann-Whitney U test revealed no effect of Diagnosis (, *p* = .433) or Presence of diagnostic criteria for apathy (p = .715) on the additional time spent playing.

**Table 2:** Performance in the attentional task.

	Total	MCI (N = 28)	Dementia (N = 29)	Apathetic (N = 23)	Non-apathetic (N = 34)
N of targets—Virtual Reality (mean ± SD)	20.6 (10.6)	23.5 (10.2)	17.8 (10.5)	19.6 (11.1)	21.3 (10.4)
N of targets—Paper	28.5 (10.9)	32.3 (11.8)	24.8 (8.7)	26.1 (1.5)	30.1 (11.7)
N. of mistakes—Virtual Reality	1.8 (1.9)	1.7 (1.5)	2.0 (2.2)	1.6 (1.5)	2.0 (2.1)
N. of mistakes—Paper	1.5 (1.4)	1.3 (1.3)	1.7 (1.4)	1.4 (1.0)	1.5 (1.5)
N of participants who continued playing—Virtual Reality	9	5	4	7	2
N of participants who continued playing—Paper	3	0	3	3	0
Additional time played—Virtual Reality	0m:41s (2m:07s)	0m:31s(1m:12s)	0m:51s (2m:44s)	0m:50s(2m:56s)	0m:35s (1m:12s)
Additional time played—Paper	0m:11s (0m:51s)	0m:0s (0m:0s)	0m:23s (1m:10s)	0m:29s (1m:18s)	0m:0s (0m:0s)

*Legend*. Number of targets found in five minutes, number of mistakes, number of participants who continued to play after the experiment, and additional time played in the VR and paper conditions for all participants (Total), participants with MCI vs. participants with dementia, and apathetic participants vs. non apathetic participants.

### Task performance

Task performance was assessed by the number of targets correctly identified, and by the number of errors (non-target character incorrectly selected) in the two conditions (see Table 2 and S1 Dataset).

A repeated-measures ANOVA with Condition (VR vs. paper) as within-subject factor and Diagnosis (dementia vs. MCI) as between subject factor revealed a main effect of Condition (F_(1,55)_ = 36.67, *p* < .001, partial η^2^ = .40) on the number of targets found, with participants (as a group) identifying significantly more targets in the paper condition (M = 28.5, SD = 10.9) compared to the VR condition (M = 20.6, SD = 10.6). In our view, this was mostly due to the low familiarity of many participants with the experimental setup, specifically the mouse, which resulted in a slow target selection. Furthermore a main effect of Diagnosis was found (F_(1,55)_ = 7.58, *p* = .008, partial η^2^ = .12), with MCI participants finding significantly more targets compared to participants with dementia. No interaction between Condition and Diagnosis was found (F_(1,55)_ = .51, *p* = .480, partial η^2^ < .01). No main effect of Condition, Diagnosis or interaction between Condition and Diagnosis was found on the number of errors (paper condition, M = 1.5, SD = 1.4; VR condition, M = 1.8, SD = 1.9; all ps > .252). Mann-Whitney U tests revealed no effect of the Presence of diagnostic criteria for apathy on the number of target founds and the errors in the VR condition, the paper condition or the difference between VR condition and paper condition (all ps > .282).

## Discussion

Virtual Reality has now emerged as a promising tool in many domains of therapy and rehabilitation, and has recently attracted the attention of researchers and clinicians working with elderly people with MCI, Alzheimer’s disease and related disorders [24]. However, due to the difficulty of working with fully-immersive VR systems in a clinical setting (e.g., equipment rarely available in the same facility where patients come for clinical consultations) very few studies have tested fully-immersive VR solutions with patients with MCI and AD [30–31]. Here we took advantage of the experience gained in a previous project where we employed VR with healthy elderly participants [33,36] and of a fully immersive VR system installed in the Nice Memory Clinic to test the feasibility of using image-based rendered VR with patients with MCI and dementia. We designed an attentional task inspired from the classical cancellation tasks [39] to train selective and sustained attention, and we tested a VR and a paper version of this task in a single-session within-subjects design.

Results of the self-reports showed that both participants with MCI and dementia were highly satisfied and interested in the attentional task, and reported high feelings of security, and low discomfort, anxiety and fatigue. In addition, participants reported to be more satisfied in the VR condition compared to the paper condition, even if the task was more difficult, as suggested by the significantly lower number of targets found in the VR condition. Accordingly, almost 70% of the participants, at the end of the task, explicitly reported to have preferred the VR condition (because it was more immersive, engaging and motivating), and among the 12 participants who decided to continue playing after the experiment, 9 did this in the VR condition. The results of the self-report questionnaires and of the additional time played are very similar for participants with MCI and dementia, thus suggesting that our VR task may be employed in patients with different levels of cognitive and functional impairment. Taken together, these findings suggest that VR can be successfully employed to make a task more interesting for elderly people with cognitive decline, possibly resulting in a higher adhesion to regular training which needs to be repeated over time to show an efficacy. Future, more controlled studies should try to disentangle which aspects of the VR task made it more interesting (e.g., the character’s animation, the 3D visualization, the big screen, etc.), so that the present results can be replicated in different trainings.

Interestingly, when exploring the ratings and time played for apathetic and non-apathetic participants, results showed that apathetic participants were as interested in the two attentional tasks as non-apathetic participants. Concerning the difference between the two conditions, apathetic participants reported to be more interested in the VR version compared to the paper version, and this preference was significantly higher compared to that of non-apathetic participants. Given that lack of interest is one of the main features of apathy [6], and that the presence of lack of interest has been shown to be a significant predictor of conversion from early MCI to AD dementia [46], designing training sessions which are interesting for apathetic participants is a key challenge in this domain, and should be considered as a clinical and research priority. Our results are in line with findings of previous studies from our group employing entertaining ICT solutions in these populations (e.g., [16]), and suggest that despite the reduction in self-initiated activities and behaviors, these patients may still be responsive to environmental-stimulated activities [6], and respond positively to them, especially if the activity meets their interests. No difference in the attentional task performance between apathetic and non-apathetic participants was found. This may be partially explained by the younger age of AD apathetic participants compared to AD non-apathetic participants (and by the significant negative correlation between age and MMSE). Future studies comparing apathetic and non-apathetic participants matched for age would be useful to ascertain whether our results can be replicated. Furthermore, the number of apathetic and non-apathetic participants was not balanced in the present study. Thus it would be important to replicate the present results with a bigger and more balanced participants’ sample.

Despite these promising results, the task still needs to be improved to fix some usability problems. The lower performance (fewer targets found) in the VR condition compared to the paper condition was mainly explained by difficulties in using the mouse to select the targets, and possibly due to eye strain from wearing the 3D glasses, and to the global VR setup, which was new to most of the participants. Many patients had never used a mouse before, and found the interaction with the mouse challenging, especially because they needed to look at the mouse while moving it. This interpretation of the results (lower performance explained by difficulties in selecting the targets, and not in finding them) should be confirmed in more controlled studies, in which the target selection phase is made more comparable across conditions. For instance, we are now working on creating a more usable user interface by employing cameras and motion capture systems (such as the Microsoft Kinect) to allow participants to select the target characters in a more naturalistic way, that is moving their arms and hands. Participants reported very low levels of anxiety, discomfort, and fatigue, and very high levels of security in the VR condition. However, although the difference was small, participants reported to feel more secure in the paper condition compared to the VR condition. This may be partially explained by the novelty of the task and of the environmental setup (mouse, 3D glasses). Future studies should test whether security feelings can be further improved by employing a more naturalistic user interface. Another limitation of the present work is that we tested the interest and satisfaction of our task in a single session, and participants were asked to play a few minutes in each condition. Thus, we cannot conclude that our training would be still interesting and satisfying after repeated, longer sessions. Results collected on the use of a serious game we recently developed to train attention and executive functions [16] suggested that motivation did not decrease after one month of extensive training. Future studies with longer and regular training sessions are needed to verify whether these findings extend to our VR task, and to verify whether regular training results in improved performance in attention and other cognitive tasks. Furthermore, before concluding that VR solutions are more interesting than classical paper-pencil solutions *per se*, a comparison between the two formats should be made employing several tasks targeting different abilities. Finally, it is important to highlight that the present study was designed in order to test the feasibility, and not the efficacy of our VR solution in enhancing performance in attention and other cognitive tasks in the target population. To test the solution efficacy, RCT should be designed including a control group of healthy elderly participants, after creating more complex versions of the task. These could be obtained, for instance, by systematically varying factors such as the number and density of the targets compared to the non-target characters, the distance of the targets from the viewer, or the similarity between target and non-target elements. Based on the results of the present feasibility study we are already designing an efficacy study, and we are starting to embed VR tasks in real rehabilitation programs.

## Supporting Information

S1 Dataset Dataset employed for the study results. (XLSX)
